# Targeted Near-Infrared Fluorescence Imaging With Iodized Indocyanine Green in Preoperative Pulmonary Localization: Comparative Efficacy, Safety, Patient Perception With Hook-Wire Localization

**DOI:** 10.3389/fonc.2021.707425

**Published:** 2021-10-27

**Authors:** Ning Ding, Kefei Wang, Jian Cao, Ge Hu, Zhiwei Wang, Zhengyu Jin

**Affiliations:** ^1^ Department of Radiology, State Key Laboratory of Complex Severe and Rare Disease, Peking Union Medical College Hospital, Chinese Academy of Medical Sciences and Peking Union Medical College, Beijing, China; ^2^ Medical Research Center, State Key Laboratory of Complex Severe and Rare Disease, Peking Union Medical College Hospital, Chinese Academy of Medical Sciences and Peking Union Medical College, Beijing, China

**Keywords:** pulmonary nodule, hook wire, indocyanine green (ICG), preoperative localization, video-assisted thoracoscopic surgery (VATS)

## Abstract

**Background:**

Precise preoperative localization is of great importance to improve the success rate and reduce the operation time of VATS surgery. This study aimed to assess the efficacy, safety, patient perception between CT-guided indocyanine green (ICG) preoperative localization of lung nodule and hook-wire localization.

**Methods:**

65 patients with 85 clinically suspicious pulmonary nodules underwent ICG preoperative localization in this study, and 92 patients with 95 nodules localized by conventional hook-wire served as controls. Both hook-wire localization and ICG injection were performed under CT guidance. Successful targeting rate, success rate in the operative field, incidence rate of complications and respiratory pain score were recorded and compared.

**Results:**

The successful targeting rate for both groups is 100%, however, due to hook-wire dislodgement, the success rate in the VATS operation field of the hook-wire group (95.6%) is lower than that of the ICG group (100%), with no significant difference(p=0.056). The overall complication rate of the hook-wire group (37.0%) is significantly higher than the ICG group (35.4%) (p=0.038). The mean respiratory pain score of the hook-wire group is 3.70 ± 1.25, which is significantly higher than that of the ICG group (2.85 ± 1.05) (p<0.001).

**Conclusions:**

ICG composed with contrast mixture are superior to the conventional hook-wire preoperative lung nodule localization procedure, with a lower complication rate, lower pain score, and relatively higher success rate. ICG is a promising alternative method for pulmonary nodule preoperative localization.

## Introduction

Lung cancer is the most common form of cancer and the leading cause of cancer death worldwide (1). Large-scale randomized clinical trials in recent years have supported the value of lung cancer mortality reduction with low-dose CT screening (2, 3).

A reasonable explanation is that detection of cancer at an earlier stage win over the opportunity for early intervention (3). For pulmonary nodule ≤ 30mm in diameter, video-assisted thoracoscopic surgery (VATS) is recommended for radical treatment with lower morbidity and shorter hospital stays compared with thoracotomy (4). However, since the location, depth of nodule, and the experience of the surgeon may vary, VATS can be changed to thoracotomy intraoperatively (5).

Several preoperative pulmonary nodule localization methods have been applied to improve the intraoperative nodule visibility (6). The most commonly adopted marking technique is perhaps the preoperative CT-guided hook-wire localization (6). The documented successful intraoperative targeting rate can be up to 94%-100% (7–9), however, complications such as pneumothorax, hemorrhage and wire dislodgement are commonly seen, while air embolism and other fetal events are rare complications. The overall complication rate of the procedure was reported around 20.1%-54% (7–10).

More importantly, it is not a comfortable experience for the patient with the hook-wire needle, a novel tumor navigation method that is less invasive is the direction for exploration.

As a novel *in vivo* tumor marking technique, indocyanine green(ICG) near-infrared (NIR) fluorescent imaging has achieved satisfactory results to enhance surgical field visualization (11–14), improve lymph node retrieval (15, 16), and exhibit endothelial abnormalities (17). Among many other dyes in the NIR spectrum, ICG is the only member introduced to clinical application (18), the wide clinical acceptance of ICG can attribute to its feature of decreased light scattering, adequate tissue penetration and low toxicity (19).

ICG has also been applied to CT-guided preoperative marking of lung nodules with safe and feasible results (20–22). Recent research has investigated the optimal ICG solution and lipiodol mixture emulsion with safe and successful out comings (23). To the best of our knowledge, little study has been focusing on the comparison between ICG and conventional hook-wire localization. This study aimed to assess the efficacy, safety, patient perception between CT-guided ICG preoperative localization of lung nodule and hook-wire localization.

## Methods

### Inclusion and Exclusion Criteria

The studies involving existing data of human participants were reviewed and approved by Peking Union Medical College Hospital Institutional Review Board. Written informed consent was obtained from all patients who received ICG or hook-wire localization. Inclusion criteria were: scheduled CT guided preoperative pulmonary nodule localization, age above 18 years, and signed informed consent. Exclusion criteria were: hemodynamic instability, pregnancy or lactation, severe coagulopathy (INR ≤ 1.5), serious cardiopulmonary dysfunction.

From October 2018 to October 2020, 92 consecutive patients with 95 nodules were localized by conventional hook-wire. The ICG was taken into practice in our institution in October 2020, after that until February 2021, 65 consecutive patients with 85 clinically suspicious pulmonary nodules who underwent ICG guided localization were included in this study.

### Computed Tomography Scan During Localization

All processes for nodular lesions localization were performed with the guidance of either NeuViz 128 CT (Neusoft Medical Company, Shenyang, China) or a dual-source CT (SOMATOM Definition, Siemens Healthcare, Germany). The scanning parameters for the location were 80 kVp tube voltage, automatically modulated tube current (reference mAs: 180), 0.6 second gantry rotation time, 1.2 pitch, 128×0.625 mm collimation width, 360 mm field of view (FOV), 1 mm slice thickness, and 1 mm slice intervals. The effective dose-length product (DLP, mGy×cm) was recorded, and the effective dose (ED, mSv) was calculated for each ablation series using the equation: ED=DLP×k·[k=0.014·(mSv×mGy^–1^×cm^–1^)].

CT was examined to distinguish the shape, size, and location of the lesion, including the interaction with the adjacent tissues and vessels with the assistance of a radio-opaque grid mesh over the chest wall. Immediately before the surgery, patients were transferred to the CT department and placed on the CT scan table in a prone, supine, or lateral decubitus position according to the location of the lesion on the CT image. CT scans were performed during a pause in normal inspiration. Images were reconstructed using a 1-mm slice thickness and a 1-mm slice interval. Then, we obtained the ideal route and marked the point of the puncture. The entry site was prepared and draped in a sterile fashion. All localization was performed under local anesthesia consisting of injection of 1% lidocaine hydrochloride.

### CT-Guided Hook-Wire Localization

**Figure 1:**
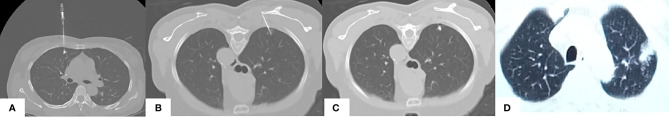
CT guided images of pulmonary nodule localization. **(A)** CT-guided injection of the iodized indocyanine green at the region of the pulmonary ground-glass opacity of a 51-year-old female. Note the supine position due to the ventral side of the nodule. **(B, C)** CT-guided hook wire localization of the pulmonary ground-glass opacity in the right upper lobe region covered by the scapula in a 62-year-old female, note the prone position due to the dorsal side of this nodule and the needle pathway is tilted to detour the scapula. **(D)** Final CT confirmed parenchymal hemorrhage in the upper lobe of the left lung in a 53-year-old male, note the emerging area of consolidation developed in the track of the puncture in the left pulmonary parenchyma.

All the localization was performed by a 7 or 10-cm-long, 20-gauge biopsy (GALLINI S.R.L., Italy) needle. The cannula needle was slowly inserted into the chest wall and lung parenchyma layer and located as close to the target nodular lesion as possible. The hook-wire was released after identifying the adequate placement of the introducer needle under the guiding CT scan, and then the introducer needle was cautiously pulled out. The examination of the CT scan was repeated to ensure the last location of the hook-wire and to search for the presence of complications such as pneumothorax or parenchymal hemorrhage (**Figure 1**). On finishing the localization procedure, patients were transferred to the pulmonary nodule VATS operation room within 60 minutes.

### CT-Guided ICG Injection

25mg indocyanine green for injection (Dandong Yichuang Pharmaceutical Co. Ltd, Liaoning, China) was mixed with 50ml of Iopamiro (Shanghai Bracco Sine Pharmaceutical Corp. Ltd., China) before positioning operation. Under the guidance of CT, the puncture point is selected according to the nodule position with the principle of “the vertical nearest”; referring to the CT images of the patient before the operation, the individualized choice of supine, prone or lateral position was chosen according to the specific nodule location for the shortest puncture pathway.

After sterilization, 5ml-10ml of 1% lidocaine was used for local infiltration anesthesia of the chest wall. After determining the direction, angle and target position, puncture the micro-puncture needle through the vicinity pathway to the pulmonary nodule. Check the CT images in between the puncture process to make sure that the needle tip is less than 15mm away from the nodule. Slowly inject 0.03 ml ICG and contrast medium mixture, and perform the final CT scan to confirm that the positioning is satisfactory, confirm that the patient’s symptom and status, and to exclude pneumothorax, bleeding, or any other complications (**Figure 1**). The pulmonary hemorrhage is defined as an emerging area of consolidation developed in the track of the puncture in the pulmonary parenchyma. The puncture point in the skin is covered with a sterile dressing, and the patient is transferred to the operating room within one hour.

### Patient Pain Score

Respiratory pain score was measured immediately after the localization procedure using the visual analog scale (PS-VAS), the patient answered the item “How do you rate your current respiratory status compared to the status before the intervention procedure?” The item had response categories on a ten-level Likert scale.

### Statistical Analysis

Patient characteristics included age, sex, height and weight, in-out localization room time, first-last CT scan interval time, pain score and complications. Nodule characteristics included the maximum diameter, vertical diameter, depth of nodule from the pleural surface, pathology. Data was analyzed using SPSS Statistical Software (SPSS for Windows, version 25.0) Continuous variables were analyzed using Student t test. Categorical variables were compared using the Chi square test or Fisher exact test. Propensity-based matching is used to select hook-wire group patients who are similar to patients receiving ICG localization method. Cox proportional hazard regression models were used to compare the adjusted complication rate and pain score between those two groups of patients.

## Results

### Hook-Wire Group

The hook-wire group of 92 patients included 26 men and 66 women, with a mean age of 53.1 ± 10.6 years. Ninety-five pulmonary nodules underwent CT-guided hook-wire localization. The computed tomography features of the 92 included patients were listed in **Table 1**. The average maximum diameter of the 95 pulmonary nodules was 6.7± 2.4 mm, with a mean distance from the pleura to the nodules of 8.4 ± 6.7 mm.

**Table 1:** Patients’ characteristics and detailed tumor parameters for both groups.

Variables	Before matching			After matching		
	Hook-wire group (No. of Patient = 92 No. of Nodule = 95)	ICG group (No. of Patient = 65 No. of Nodule = 85)	P value	Hook-wire group (No. of Patient = 64 No. of Nodule = 65)	ICG group (No. of Patient = 64 No. of Nodule = 65)	P value
Age, years	53.1 ± 10.6	51.3 ± 11.6	0.299	51.9 ± 11.1	51.3 ± 11.7	0.781
Gender			0.611			0.676
Male	26	16		14	16	
Female	66	49		50	48	
Height (m)	1.65 ± 0.07	1.65 ± 0.07	0.617	1.65 ± 0.07	1.65 ± 0.07	0.834
Weight (kg)	63.4 ± 10.5	63.8 ± 11.7	0.836	63.4 ± 10.7	63.4 ± 11.4	0.997
Localization duration (Time between in-out the localization room, min)	24.47 ± 2.46	24.52 ± 3.43	0.911	24.19 ± 2.35	24.53 ± 3.46	0.512
Localization duration (Time between first-last CT scan, min)	11.22 ± 1.91	11.34 ± 3.08	0.779	11.30 ± 1.87	11.38 ± 3.09	0.863
Maximum nodule diameter (mm)	6.7 ± 2.4	6.3 ± 2.4	0.209	6.6 ± 2.1	6.4 ± 2.4	0.655
Vertical nodule diameter (mm)	5.3 ± 2.6	4.6 ± 1.9	0.039	5.0 ± 1.8	4.8 ± 1.9	0.603
Depth of nodule from surface (mm)	8.4 ± 6.7	9.2 ± 10.0	0.528	8.4 ± 6.4	8.2 ± 9.0	0.881
Nodule location			0.127			0.999
Right upper lobe	23 (24.2%)	33 (38.8%)		20 (30.8%)	20 (30.8%)	
Right middle lobe	12 (12.6%)	11 (12.9%)		9 (13.8%)	9 (13.8%)	
Right lower lobe	19 (20.0%)	19 (22.4%)		16 (24.6%)	16 (24.6%)	
Left upper lobe	17 (17.9%)	8 (9.4%)		7 (10.8%)	8 (12.3%)	
Left lower lobe	24 (25.3%)	14 (16.5%)		13 (20.0%)	12 (18.5%)	
Radiological pattern			0.942			0.427
Solid nodule	13 (13.7%)	13 (15.3%)		9 (13.8%)	7 (10.8%)	
Part-solid nodule	32 (33.7%)	29 (34.1%)		19 (29.2%)	26 (40.0%)	
Pure ground glass nodule	50 (52.6%)	43 (50.6%)		37 (56.9%)	32 (49.2%)	
Pathological diagnosis			0.154			0.589
Benign lesions	17 (17.9%)	12 (14.1%)		10 (15.4%)	10 (15.4%)	
Granuloma	0 (0%)	1 (1.2%)		0 (0%)	1 (1.5%)	
Atypical adenomatous hyperplasia primary	10 (10.5%)	5 (5.9%)		7 (10.8%)	5 (7.7%)	
Adenocarcinoma *in situ*	21 (22.1%)	22 (25.9%)		15 (23.1%)	21 (32.3%)	
Minimally invasive adenocarcinoma	16 (16.8%)	23 (27.1%)		13 (20.0%)	14 (21.5%)	
Invasive adenocarcinoma	31 (32.6%)	19 (22.4%)		20 (30.8%)	13 (20.0%)	
Hamartoma	0	1 (1.2%)		0 (0%)	0 (0%)	
Bronchial adenoma	0	2 (2.4%)		0 (0%)	1 (1.5%)	

The targeting success rate after the final CT scan of the hook-wire localization was 100% of 95 nodules. However, the successful localization in the operative field is 95.6%, since the hook-wire was dislodged or fallen out before VATS resection in four targeted nodular lesions. However, they were successfully resected with the assistance of the remnant hemorrhagic marks on the lung parenchymal surface arising from the procedure of localization. The most common complication related to hook-wire localization was pneumothorax (n=26, 28.3%), diagnosed by CT scanning right after the localization procedure. The other complication is lung parenchymal hemorrhage (n=8, 8.7%). However, all patients with pneumothorax and hemorrhage were asymptomatic, and no further intervention procedure was required before operation. Hypotension occurred in one case after the operation. It was considered as the pleural reaction, and recovery was achieved with fluid supplementation and supportive treatment. The surgical margins were all negative on final pathology in all included cases. The details of the hook-wire localization procedure are shown in **Table 1**.

Histologic diagnosis included inflammatory benign lesions(n=17), atypical adenomatous hyperplasia primary (n=10), adenocarcinoma *in situ* (n=21), minimally invasive adenocarcinoma (n=16), invasive adenocarcinoma (n=31). **Table 1** represents the details of pathologic results.

### ICG Group

65 patients were included, among them, 16 men and 49 women, with a mean age of 51.3 ± 11.6 years. In total, 85 pulmonary nodules underwent CT-guided ICG localization. The computed tomography features of the 65 included patients were listed in **Table 1**. The average maximum diameter of the pulmonary nodules was 6.3 ± 2.4mm, with a mean distance from the pleura to the nodules of 9.2 ± 10.0 mm.

The targeting success rate of ICG localization was 100% of 85 nodules. Moreover, the successful localization in the operative field is 100%. The most common complication related to ICG localization was pneumothorax (n=18, 27.7%), diagnosed by the immediate CT scanning right after the localization procedure. The other complication is lung parenchymal hemorrhage (n=5, 7.7%). However, all patients with pneumothorax and hemorrhage were asymptomatic, and no further interventional procedure was required before operation. The surgical margins were all negative on final pathology in all included cases. The details of the ICG localization procedure are shown in **Table 1**.

Histologic diagnosis for included inflammatory benign lesions (n=12), granuloma (n=1), atypical adenomatous hyperplasia primary (n=5),adenocarcinoma *in situ* (n=22),minimally invasive adenocarcinoma (n=23), invasive adenocarcinoma (n=19),hamartoma (n=1),bronchial adenoma(n=2). **Table 1** represents the details of pathologic results.

### Propensity-Score Based Balancing

The basic characteristic data of the two groups showed no significant difference except the vertical nodule diameter. In order to obtain more accurate results and reduce potential selection bias, we performed propensity matching for the two groups, the nearest neighbor matching method is adopted. The match tolerance was 0.02. A total of 64 subjects were matched with 65 nodules after conducting the propensity analysis. There was no statistical difference between all feature data after the matching, the details features before and after the propensity score matching are listed in **Table 1**.

### Comparison Between ICG and Hook-Wire Preoperative Localization

The successful targeting rate for both groups is 100%, however, due to hook-wire dislodgement, the success rate in the VATS operation field of the hook-wire group (95.6%) is lower than that of the ICG group (100%), with no significant difference. The overall complication rate of the hook-wire group (37.0%) is significantly higher than the ICG group (35.4%) (p=0.038). The mean respiratory pain score of the hook-wire group is 3.70 ± 1.25, which is significantly higher than that of the ICG group (2.85 ± 1.05) (=<0.001). (**Table 2**)

**Table 2:** Efficacy, safety and patient perception of CT guided ICG preoperative localization of lung nodule and hook-wire localization.

variables	Hook-wire (n = 92)	ICG group (n = 65)	P value
Successful targeting rate	100%	100%	NA
Success rate in the operative field	95.6% (88/92)	100% (65/65)	0.056
Incidence rate of complications	37.0% (34/92)	35.4% (23/65)	0.038
Respiratory pain score	2.85 ± 1.05	3.70 ± 1.25	<0.001

NA, Not Available.

## Discussion

Identifying non-palpable and non-visible pulmonary nodule during VATS surgery is challenging, precise preoperative localization is of great importance to improve the success rate and reduce operation time of VATS surgery. Accordingly, various preoperative localization methods have been applied and studied to assist VATS to reduce operative difficulty and time, each kind of method has its own advantages and shortcomings, among them, hook-wire is the most widely applied method (7–10, 24).To the best of our knowledge, this study is the first comparative study between conventional hook-wire and emerging methods of ICG in the comprehensive perspective of efficacy, safety, and patient perception.

One recently published meta-analysis including 46 studies by Chul Hwan Park et al. compared the efficacy and safety of three preoperative methods (6), the reported success rate of hook-wire localization is about 98%. However, the successful localization rate in the operative field is around 94% with significant heterogeneity. Mark Kleedehn and colleagues have performed the study comparing methylene blue, another dye marker method, with hook-wire techniques, their study showed that the methylene blue method group achieved an equivalent preoperative localization success rate with a relatively lower complication percentage, this was similar to our finding (24).In our study, the successful targeting rate right after the localization is 100%, however, four hook-wires migrated or dislodged before or during the VATS operation, even though the surgeon managed to resect the nodules in the end, considering the prolonged VATS operation time and the extra procedures to handle the related hemorrhage, these four cases was classified as a failure in the term of intraoperative successful rate in this study. Therefore, the success rate of the hook-wire group in the operation field is 95.6%, which is consistent with the aforementioned systematic review.

In the ICG group, all 85 nodules were identified under the intraoperative near-infrared ray (NIR) thoracoscope to visualize ICG fluorescence, both the targeting success rate and the intraoperative successful rate was 100%, this result is consistent with early clinical studies with relatively fewer cases (n=30-37) applying ICG in pulmonary nodule preoperative localization (20–22). Even though studies have shown that ICG tends to be diffused easily (23), which may cause over resection of peritumor tissue, the actual period of ICG *in vivo* is unknown, one case report has shown the existence of ICG even 6 days after injection under infrared thoracoscopy (25). More researches are needed to explicate this issue.

**Figure 2:**
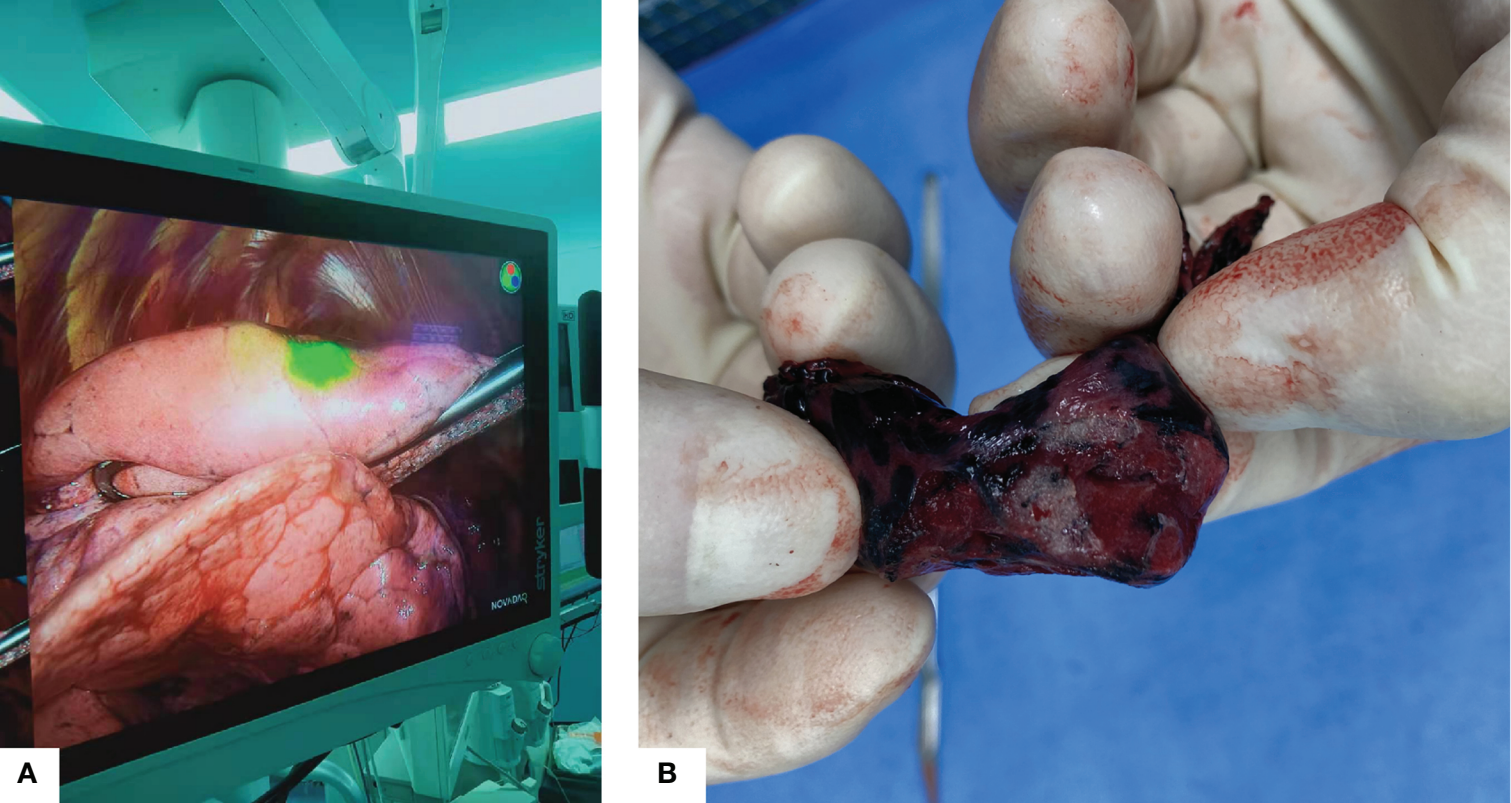
**(A)** Intraoperative fluorescence using near-infrared thoracoscope, the nodule was localized in the green light area. **(B)** Gross appearance of the resected specimen.

One innovative point of the current study is that we have taken measures to deal with this problem, firstly, the preoperative localization procedure was scheduled within one hour before the VATS surgery, regardless hook-wire or ICG was used. Also, the mixed ICG and iodine solution was applied to reduce diffusivity (23). In the VATS operative field, ICG was rather localized than diffused, focused in the area where the nodule can be easily detected (**Figure 2**).

The difference was noted of the average distance from the pleura to the nodules tends to be greater in the ICG group (9.2 ± 10.0mm in the ICG group *vs.* 8.4 ± 6.7 mm), however, the difference showed no statistical significance(p=0.528) even before the propensity analysis. As mentioned in the method section, the needle tip was placed less than 15mm away from the nodule to inject the solution. However, in the hook-wire group, the tip of the wire is placed as close to the target lesion as possible. Therefore, the ICG technique is prone to be applied in deeper localized lesion compared with the hook-wire arm. In another way, an underlying reason for the dislodgement in the hook-wire arm may result from more superficial lesions being localized and this should be mentioned in the discussion.

In terms of safety, any kind of complications in both groups were recorded, pneumothorax and hemorrhage were frequently seen in both groups, however, there is no significant difference regarding the incidence rate of these complications.

Pain is a major concern for human beings, another innovative point of this study is that we included the patient perception survey into consideration. The chief complaint after preoperative localization is breath pain. Based on this, the patient perception of each procedure was quantified by the VAS score. The hook-wire group showed a significantly higher pain score than the ICG group(p<0.001). This can be easily understood, given the rigid hook-wire nature into account.

The limitation of our study is obvious, as it is a single-center retrospective study. The sample size is relatively small and the study’s design was not a randomized controlled trial, even though a propensity-score based balancing procedure is performed to reduce selection bias, the robust of feasibility and safety can be inhomogeneous in different settings. Multicenter RCT research is needed to further study the accuracy of ICG localization technology. Second, the size and depth of nodule can influence the success rate of localization procedure and following VATS surgery. Based on the few failure cases, we did not provide evidence between the size, location of the nodule, and the success rate. A larger cohort is needed to analyze the potential relationship and provide a reference for the surgeons to make individualized surgery plan.

## Conclusions

The current study suggests that the ICG composed with contrast mixture is superior to the conventional hook-wire preoperative lung nodule localization procedure, with a lower complication rate, lower pain score, and relatively higher success rate. ICG can be applied as a promising alternative method for the pulmonary nodule preoperative localization.

## Data Availability Statement

The original contributions presented in the study are included in the article/supplementary material. Further inquiries can be directed to the corresponding authors.

## Ethics Statement

The studies involving existing data of human participants were reviewed and approved by Peking Union Medical College Hospital Institutional Review Board. Written informed consent was obtained from all patients who received ICG or hook wire localization.

## Author Contributions

All the authors conceived and designed the experiments. KW, JC and ZW conducted experiments. ND, GH and ZW prepared the figures and tables. ND, GH analyzed the data and wrote the manuscript. All the authors reviewed and revised the manuscript. All authors read and approved the manuscript.

## Funding

This reaearch was funded by the Tsinghua University-Peking Union Medical College Hospital Initiative Scientific Research Program (20191080922).

## Conflict of Interest

The authors declare that the research was conducted in the absence of any commercial or financial relationships that could be construed as a potential conflict of interest.

## Publisher’s Note

All claims expressed in this article are solely those of the authors and do not necessarily represent those of their affiliated organizations, or those of the publisher, the editors and the reviewers. Any product that may be evaluated in this article, or claim that may be made by its manufacturer, is not guaranteed or endorsed by the publisher.

## References

[1] Bray F Ferlay J Soerjomataram I Siegel RL Torre LA Jemal A. Global Cancer Statistics 2018: GLOBOCAN Estimates of Incidence and Mortality Worldwide for 36 Cancers in 185 Countries. CA Cancer J Clin (2018) 68:394–424. doi: 10.3322/caac.21492 30207593

[2] de Koning HJ van der Aalst CM de Jong PA Scholten ET Nackaerts K Heuvelmans MA. Reduced Lung-Cancer Mortality With Volume CT Screening in a Randomized Trial. N Engl J Med (2020) 382:503–13. doi: 10.1056/NEJMoa1911793 31995683

[3] Aberle DR Adams AM Berg CD Black WC Clapp JD Fagerstrom RM. Reduced Lung-Cancer Mortality With Low-Dose Computed Tomographic Screening. N Engl J Med (2011) 365:395–409. doi: 10.1056/NEJMoa1102873 21714641 PMC4356534

[4] Ost D Fein AM Feinsilver SH. Clinical Practice. The Solitary Pulmonary Nodule. N Engl J Med (2003) 348:2535–42. doi: 10.1056/NEJMcp012290 12815140

[5] Grills IS Mangona VS Welsh R Chmielewski G McInerney E Martin S. Outcomes After Stereotactic Lung Radiotherapy or Wedge Resection for Stage I non-Small-Cell Lung Cancer. J Clin Oncol (2010) 28:928–35. doi: 10.1200/JCO.2009.25.0928 20065181

[6] Park CH Han K Hur J Lee SM Lee JW Hwang SH. Comparative Effectiveness and Safety of Preoperative Lung Localization for Pulmonary Nodules: A Systematic Review and Meta-Analysis. Chest (2017) 151:316–28. doi: 10.1016/j.chest.2016.09.017 27717643

[7] Rostambeigi N Scanlon P Flanagan S Frank N Talaie R Andrade R. CT Fluoroscopic-Guided Coil Localization of Lung Nodules Prior to Video-Assisted Thoracoscopic Surgical Resection Reduces Complications Compared to Hook-Wire Localization. J Vasc Interv Radiol (2019) 30:453–9. doi: 10.1016/j.jvir.2018.10.013 30819493

[8] Park JB Lee SA Lee WS Kim YH Song I Lee JG. Computed Tomography-Guided Percutaneous Hook-Wire Localization of Pulmonary Nodular Lesions Before Video-Assisted Thoracoscopic Surgery: Highlighting Technical Aspects. Ann Thorac Med (2019) 14:205–12. doi: 10.4103/atm.ATM_287_18 PMC6611205 31333771

[9] Li C Liu B Jia H Dong Z Meng H. Computed Tomography-Guided Hook-Wire Localization Facilitates Video-Assisted Thoracoscopic Surgery of Pulmonary Ground-Glass Nodules. Thorac Cancer (2018) 9:1145–50. doi: 10.1111/1759-7714.12801 PMC6119612 30047619

[10] Ciriaco P Negri G Puglisi A Nicoletti R Del Maschio A Zannini PJE. Video-Assisted Thoracoscopic Surgery for Pulmonary Nodules: Rationale for Preoperative Computed Tomography-Guided Hookwire Localization. Eur J Cardiothorac Surg (2004) 25:429–33. doi: 10.1016/j.ejcts.2003.11.036 15019673

[11] Nicoli F Saleh D Baljer B Chan C Beckingsale T Ghosh K. Intraoperative Near-Infrared Fluorescence (NIR) Imaging With Indocyanine Green (ICG) Can Identify Bone and Soft Tissue Sarcomas Which May Provide Guidance for Oncological Resection. Ann Surg (2021) 273:e63–8. doi: 10.1097/SLA.0000000000003857 32224746

[12] Nishino H Hatano E Seo S Nitta T Saito T Nakamura M. Real-Time Navigation for Liver Surgery Using Projection Mapping With Indocyanine Green Fluorescence: Development of the Novel Medical Imaging Projection System. Ann Surg (2018) 267:1134–40. doi: 10.1097/SLA.0000000000002172 28181939

[13] Lee Z Moore B Giusto L Eun DD. Use of Indocyanine Green During Robot-Assisted Ureteral Reconstructions. Eur Urol (2015) 67:291–8. doi: 10.1016/j.eururo.2014.08.057 25220372

[14] Siddighi S Yune JJ Hardesty J. Indocyanine Green for Intraoperative Localization of Ureter. Am J Obstet Gynecol (2014) 211:436.e1–2. doi: 10.1016/j.ajog.2014.05.017 24835212

[15] Chen QY Xie JW Zhong Q Wang JB Lin JX Lu J. Safety and Efficacy of Indocyanine Green Tracer-Guided Lymph Node Dissection During Laparoscopic Radical Gastrectomy in Patients With Gastric Cancer: A Randomized Clinical Trial. JAMA Surg (2020) 155:300–11. doi: 10.1001/jamasurg.2019.6033 32101269

[16] Yuan L Qi X Zhang Y Yang X Zhang F Fan L. Comparison of Sentinel Lymph Node Detection Performances Using Blue Dye in Conjunction With Indocyanine Green or Radioisotope in Breast Cancer Patients: A Prospective Single-Center Randomized Study. Cancer Biol Med (2018) 15:452–60. doi: 10.20892/j.issn.2095-3941.2018.0270 PMC6372915 30766755

[17] Verjans JW Osborn EA Ughi GJ Calfon Press MA Hamidi E Antoniadis AP. Targeted Near-Infrared Fluorescence Imaging of Atherosclerosis: Clinical and Intracoronary Evaluation of Indocyanine Green. JACC Cardiovasc Imaging (2016) 9:1087–95. doi: 10.1016/j.jcmg.2016.01.034 PMC5136528 27544892

[18] Pauli J Brehm R Spieles M Kaiser W Hilger I Resch-Genger UJJ. Novel Fluorophores as Building Blocks for Optical Probes for *In Vivo* Near Infrared Fluorescence (NIRF) Imaging. J Fluoresc (2010) 20:681–93. doi: 10.1007/s10895-010-0603-7 20213244

[19] Porcu EP Salis A Gavini E Rassu G Maestri M Giunchedi P. Indocyanine Green Delivery Systems for Tumour Detection and Treatments. Biotechnol Adv (2016) 34:768–89. doi: 10.1016/j.biotechadv.2016.04.001 27090752

[20] Zhong L Hu W Li S Wei Z Zhu Z Jin G. Clinical Study of Video-Assisted Thoracoscopic Surgery Wedge Resection in Early-Stage Lung Cancer by Tumor Mapping With Indocyanine Green. Wideochir Inne Tech Maloinwazyjne (2019) 14:545–50. doi: 10.5114/wiitm.2019.89986 PMC6939215 31908701

[21] Zhang C Lin H Fu R Zhang T Nie Q Dong S. Application of Indocyanine Green Fluorescence for Precision Sublobar Resection. Thorac Cancer (2019) 10:624–30. doi: 10.1111/1759-7714.12972 PMC6449268 30734507

[22] Nagai K Kuriyama K Inoue A Yoshida Y Takami K. Computed Tomography-Guided Preoperative Localization of Small Lung Nodules With Indocyanine Green. Acta Radiol (2018) 59:830–5. doi: 10.1177/0284185117733507 28971708

[23] Rho J Lee JW Quan YH Choi BH Shin BK Han KN. Fluorescent and Iodized Emulsion for Preoperative Localization of Pulmonary Nodules. Ann Surg (2021) 273(5):989–96. doi: 10.1097/SLA.0000000000003300 30973387

[24] Kleedehn M Kim DH Lee FT Lubner MG Robbins JB Ziemlewicz TJ. Preoperative Pulmonary Nodule Localization: A Comparison of Methylene Blue and Hookwire Techniques. AJR Am J Roentgenol (2016) 207:1334–9. doi: 10.2214/AJR.16.16272 27657546

[25] Li X Zeng Y Liu J Cui F He J. Indocyanine Green Remains in the Lung for Up to 6 Days. Ann Thorac Surg (2020) 110:e385–6. doi: 10.1016/j.athoracsur.2020.03.076 32360873

